# Editorial: Sleep and circadian rhythms in cancer patients and their relationship with quality of life

**DOI:** 10.3389/fnins.2022.1060184

**Published:** 2022-11-28

**Authors:** Joy Perrier, Bénédicte Giffard, Lisa M. Wu, Josée Savard, Ali Amidi

**Affiliations:** ^1^Normandie Université, UNICAEN, PSL Université, EPHE, INSERM, U1077, CHU de Caen, GIP Cyceron, Neuropsychologie et Imagerie de la Mémoire Humaine, Caen, France; ^2^Cancer and Cognition Platform, Ligue Nationale Contre le Cancer, Caen, France; ^3^Department of Medical Social Sciences, Northwestern University Feinberg School of Medicine, Chicago, IL, United States; ^4^Aarhus Institute of Advanced Studies, Aarhus University, Aarhus, Denmark; ^5^Sleep & Circadian Psychology Research Group, Department of Psychology and Behavioural Sciences, Aarhus University, Aarhus, Denmark; ^6^School of Psychology, Université Laval, Quebec City, QC, Canada; ^7^CHU de Québec-Université Laval Research Center, Quebec City, QC, Canada; ^8^Laval University Cancer Research Center, Quebec City, QC, Canada

**Keywords:** cancer, sleep, cognition, fatigue, anxio-depressive factors

We spend around one third of our lives sleeping and a good night of sleep is critical for many important functions such as learning and memory, brain restoration and plasticity, and immune functioning. Furthermore, inadequate sleep and insomnia are linked to numerous illnesses including metabolic and cardiovascular diseases. Cancer patients have frequent complaints of insomnia, with a high prevalence of 30–60% before, during and after treatment (Savard et al., 2011). There is also emerging evidence of circadian disruption associated with cancer and cancer treatments, which may underlie and exacerbate commonly reported symptoms of depressed mood, fatigue, cognitive complaints and sleep disturbances (ANSES, 2016; Labrèche et al., 2019; Martin et al., 2021; Milanti et al., 2021; Amidi and Wu, 2022). Despite the important impact of sleep on mental and physical health, until recently, sleep disturbances following cancer and its treatment were often neglected by both clinicians and researchers.

This Research Topic aims to elucidate sleep and circadian rhythm disturbances associated with cancer and cancer treatments, as well as their impact on quality of life and survivorship. The high quality of the manuscripts published in this Research Topic highlights the increased interest in the field of sleep/circadian rhythms and cancer and the broad scope of work in the area. Three reviews highlighted the importance of sleep and circadian rhythms in cancer patients as potential drivers of quality-of-life sequelae, five papers investigated quality of life and sleep and circadian rhythm biomarkers in cancer patients and, two papers focused on innovative approaches to tackle sleep and circadian rhythm alterations in cancer patients.

## Sleep disruption and cancer: Beyond quality of life

The first review perfectly exemplifies the current debate in the scientific literature about sleep and cancer with the so-called “chicken or the egg” question. Berisha et al. present an elegant review that discusses existing evidence about sleep disruption both as a consequence or as a potential risk factor of cancer. After describing sleep disruption-induced changes in systemic physiology and associated changes in the central nervous system, the authors explain the clinical implications of these findings, as well as the need to better manage sleep disruption in cancer patients, such as through the use of melatonin. Finally, unanswered questions and future directions are discussed in order to pave the way for future studies.

Two more reviews give additional insights into the same “chicken or the egg” question, but are not able to definitively conclude that a causal relationship exists between sleep disruption and quality of life/prognosis outcomes. The systematic review and meta-analysis by Strom et al. focuses on associations between sleep/sleep-wake activity and prognostic outcomes in cancer patients. Among the 26 studies included in their review, 19 report associations between poorer sleep, and a poorer response to treatment, shorter time to progression, and/or reduced overall survival. However, the authors also highlight several limitations of the studies reviewed, such as the high heterogeneity of sleep measures across studies that weaken comparability, and the small number of studies that used objective measures of sleep with the remainder relying primarily on single-item questionnaires. The authors conclude that disturbed sleep during treatment may be a relevant behavioral marker of poor cancer prognosis that warrants further investigation.

The systematic review by Helligsoe et al. describes the nature of sleep disorders in survivors of childhood central nervous system tumor and explores the association between tumor location and diagnosed sleep disorder. Only 11 studies are included in the review, pointing to the scarcity of studies that have objectively assessed sleep disorders using polysomnography with proper diagnosis in brain tumor patients. Analyses show that sleep disorders are common among children who have survived a central nervous system tumor with the most common being sleep-related breathing disorders (i.e., obstructive sleep apnea) and central disorders of hypersomnolence (i.e., narcolepsy).

## Links between sleep/circadian rhythm dysregulation and quality of life in cancer

Four papers examine associations between sleep and sleep-wake rhythms and aspects of cognitive functioning and quality of life. Trivedi et al. investigate post-treatment sleep-wake rhythm outcomes using actigraphy in non-central nervous system cancer (including endometrial and breast cancer and melanoma). Results show that greater sleep regularity is associated with better quality of life and better physical functioning highlighting the need for interventions to help maintain sleep-wake rhythm regularity in cancer patients. Using a large cohort of patients, Garland et al. investigate longitudinal associations between insomnia symptoms and perceived cognitive impairments in cancer patients using validated questionnaires. Results show that the presence of both insomnia and perceived cognitive impairments is significantly greater within 2 months after surgery than later on during treatment. The authors also identify important factors that are related to a higher risk of reporting comorbid insomnia and cognitive impairment. Duivon et al.'s study investigates prospective memory, i.e., the memory for future intentions in breast cancer patients treated with surgery, radiotherapy followed or not by endocrine therapy. They detect subtle changes in cortical activity (EEG) related to memory consolidation during sleep but that are not related to memory performance itself. In the study by Olsthoorn et al., caregivers of 83 pediatric brain tumor survivors were surveyed about their children's sleep difficulties. Results show that at least one sleep-related item was scored as “somewhat true” for 68% of the children and a higher level of sleep disturbance is related to worse sluggish cognitive tempo (i.e., confusion, slowed behavior and low motivation). Finally, Oliva et al. focus on inflammatory biomarkers, as assessed with plasma samples, and their relationships with self-reported sleep quality in cancer patients both before and during oncological treatments. Results indicate that, across treatments, higher sleep complaints are associated with increased levels of a number of pro-inflammatory biomarkers 3 months after treatment initiation. Authors emphasize the need to evaluate biomarkers and self-reported sleep at multiple time-points across treatment instead of single point measurements.

## Targeting sleep/circadian rhythm dysregulation in cancer patients

Two intervention trials are presented that aim to improve sleep and circadian rhythm disruption in cancer patients. These studies highlight movement in the field toward prevention and regulation of disrupted sleep and circadian rhythms in cancer patients to improve quality of life and prognostic outcomes. One study is a Phase II randomized controlled trial by Rissling et al. in which they test whether morning exposure to bright white light would maintain or improve sleep and circadian activity rhythms in breast cancer patients undergoing chemotherapy when compared with a dim red light comparison condition. Results show that morning exposure to bright light is associated with longer night-time sleep, fewer sleep disturbances, fewer and shorter daytime naps, and less activity at night and more activity during the day by the end of cycle 4 of chemotherapy. These results show promise for the use of bright light therapy in cancer patients in order to reduce sleep and circadian rhythms disturbances, although larger scale trials are still necessary. Finally, Fox et al. present a study protocol focused on gynecologic cancer patients, a patient population that has been neglected in sleep studies as compared with breast cancer patients. The authors propose a two-part protocol guided by the Multiphase Optimization Strategy (MOST) framework. The first part will serve to identify barriers to and facilitators of intervention adherence. In the second part of the protocol, that will test the efficacy of the intervention, participants will be randomized to one of eight conditions (i.e., stimulus control, sleep restriction and bright light therapy either alone or in combination) and will complete assessments from baseline up to 3 months post-intervention. Measurements will include subjective and objective (i.e., actigraphy) sleep measures as well as questionnaires related to symptom burden, quality of life, fatigue and urine samples to analyze urinary 6-sulfatoxymelatonin, the primary urinary metabolite of melatonin. The ultimate goal of this study is to propose a framework to develop an efficient and effective, minimally burdensome behavioral sleep intervention.

## Conclusion

Studies published in this Research Topic clearly underscore the significance of sleep and circadian rhythms disruptions in cancer patients and their possible impact on quality of life and perhaps even prognostic outcomes. Future research would benefit from deeper and integrative investigations of sleep and circadian rhythms over longer periods. Indeed, although actigraphy can provide information about rest-activity rhythms, it can be influenced by many parameters, including the quality of sleep and daytime sleepiness, and by environmental factors such as light exposure. It is thus, highly suggested to evaluate sleep and circadian processes in a multimodal manner that, in addition to questionnaires and actigraphy, includes the assessment of circadian physiology and other parameters (e.g., dim light melatonin onset, light exposure, and sleep hygiene behaviors). For instance, the use of polysomnography (when possible) would help better understanding changes to sleep architecture that occur in cancer patients. Moreover, using polysomnography would facilitate the study of memory consolidation during sleep, known to be altered during wake in cancer (Perrier et al., 2020; Duivon et al., 2021). Moreover, additional studies of sleep and circadian rhythms are needed in relation to cancer treatments beyond chemotherapy, such as endocrine- and immune therapies, with the former being proposed to most breast cancer patients for at least 5 years and that may also impact cognitive functioning (Wu and Amidi, 2017). Finally, there is an increasing number of interventional studies targeting sleep problems or aiming to improve circadian rhythms in cancer patients being published (Garland et al., 2014; Wu et al., 2018, 2022; Savard et al., 2022). Future investigations will hopefully determine the optimal approach to administer cancer treatments informed by circadian biology, such as tailoring the timing of treatments to individuals' chronotype (e.g., Innominato et al., 2022; Printezi et al., 2022).

## Author contributions

JP wrote the first draft of the manuscript. BG, LW, JS, and AA provide revisions and comments. All authors contributed to the article and approved the submitted version.

## Funding

JP's and BG's work was supported by the ARC Foundation—for cancer research (2017–2020), the French Sleep Society (SFRMS), the Région Normandie (Réseaux d'Intérêts Normands, RIN), the Cancéropôle Nord-Ouest, and the Ligue Nationale Contre le Cancer. LW's effort was supported by the American Cancer Society Award Number 131642-RSG-18-053-01-PCSM, and the European Union Horizon 2020 Research and Innovation Program under the Marie Sklodowska-Curie Grant Agreement No. 754513 and the Aarhus University Research Foundation. AA's effort was supported by the Danish Cancer Society (R174-A11447-17-S52).

## Conflict of interest

The authors declare that the research was conducted in the absence of any commercial or financial relationships that could be construed as a potential conflict of interest.

## Publisher's note

All claims expressed in this article are solely those of the authors and do not necessarily represent those of their affiliated organizations, or those of the publisher, the editors and the reviewers. Any product that may be evaluated in this article, or claim that may be made by its manufacturer, is not guaranteed or endorsed by the publisher.
